# Knockdown of FRAT1 Expression by RNA Interference Inhibits Human Glioblastoma Cell Growth, Migration and Invasion

**DOI:** 10.1371/journal.pone.0061206

**Published:** 2013-04-17

**Authors:** Geng Guo, Dong Kuai, Sang Cai, Naizhao Xue, Yueting Liu, Jiehe Hao, Yimin Fan, Ji Jin, Xinggang Mao, Bolin Liu, Chengliang Zhong, Xiang Zhang, Yi Yue, Xiaodong Liu, Ning Ma, Yuhong Guo

**Affiliations:** 1 Department of Neurosurgery, The First Hospital, Shanxi Medical University, Taiyuan, Shanxi Province, People's Republic of China; 2 Institute of Neurosurgery, No.101 Hospital of PLA, Wuxi, Jiangsu Province, People's Republic of China; 3 Shanxi Medical University, Taiyuan, Shanxi Province, People's Republic of China; 4 Department of Neurosurgery, PLA 254 Hospital, Tianjin, People's Republic of China; 5 Department of Neurosurgery, Xijing Hospital, Fourth Military Medical University, Xi’an, Shaanxi Province, People's Republic of China; 6 Clinical Pharmacological Center, The First Teaching Hospital of Tianjin University of Traditional Chinese Medicine, Tianjin, People's Republic of China; 7 Department of Neurosurgery, Shanxi’s General Hospital of Chinese People’s Armed Police Force, Taiyuan, Shanxi Province, People's Republic of China; University of Florida, United States of America

## Abstract

**Background:**

FRAT1 positively regulates the Wnt/β-catenin signaling pathway by inhibiting GSK-3-mediated phosphorylation of β-catenin. It was originally characterized as a protein frequently rearranged in advanced T cell lymphoma, but has recently also been identified as a proto-oncogene involved in tumorigenesis. Our previous studies showed that FRAT1 was dramatically overexpressed in gliomas and its expression level was significantly increased along with clinicopathological grades.

**Methods:**

In the current study, we used RT-PCR and Western blotting to assess the mRNA and protein levels of FRAT1 in three glioma cell lines. In addition, to evaluate its functional role in gliomas, we examined the effects of FRAT1 knockdown on proliferation, migration and invasion in vitro and tumor growth in vivo using glioblastoma U251 cells and RNAi.

**Results:**

FRAT1 was highly expressed in all three glioma cell lines. RNAi-mediated down-regulation of endogenous FRAT1 in human glioblastoma U251 cells resulted in suppression of cell proliferation, arrest of cell cycle, inhibition of cell migration and invasion in vitro. Moreover, FRAT1 depletion significantly impaired tumor xenograft growth in nude mice.

**Conclusions:**

Our results highlight the potential role of FRAT1 in tumorigenesis and progression of glioblastoma. These findings provide a biological basis for FRAT1 as a potential molecular marker for improved pathological grading and as a novel candidate therapeutic target for glioblastoma management.

## Introduction

Glioblastoma is the most common and lethal type of primary central nervous system neoplasm in adults. Although the comprehensive treatment strategy for glioblastomas is continuously progressing, the outcome of this malignancy is still very poor. Patients with glioblastoma carry extremely poor prognosis, with a median survival period of about 1 year, despite surgical resection combined with radiotherapy and chemotherapy [1], [2]. Challenges concerning treatment are associated closely with the inherent biologic properties of the glioblastoma, such as excessive proliferation and relentless invasion. Therefore, in order to improve the current therapeutic regimens, it is necessary to better understand the molecular mechanisms involved in the uncontrolled proliferation and invasion of glioblastomas, and to identify specific biomarkers in tumorigenesis associated with progression of this malignancy.

The FRAT1 (frequently rearranged in advanced T-cell lymphomas-1) gene, located on human chromosome 10q24.1 [3], encodes a 29-kDa protein comprising 279 amino acids. FRAT1 has been identified as a positive regulator of the Wnt/β-catenin pathway, which can inhibit the GSK-3-mediated phosphorylation of β-catenin [4], [5], [6]. Currently, accumulating evidence demonstrates that FRAT1 plays a role in tumor progression [7], [8], [9], [10], [11], [12]. Our previous study showed that aberrant expression of FRAT1 is significantly correlated with the pathologic grade and tumor proliferation rate in surgically resected glioma tissues, implying an oncogenic role for FRAT1 in gliomagenesis [13], [14]. However, the expression of FRAT1 in specific glioma cell lines has not been elucidated. In the present study, we investigated FRAT1 expression levels in three established glioma cell lines (U87, U251 and SHG44). Moreover, we explored the role of FRAT1 in the proliferation, migration and invasion of U251 glioblastoma cells in vitro and in vivo by knocking-down FRAT1 with RNA interference (RNAi). These results provide further insight into the role of FRAT1, and increase the understanding of the biological basis of glioblastoma by demonstrating the potential of FRAT1 as a prognostic biomarker and therapeutic target in clinical application.

## Materials and Methods

### Cell Lines and Cell Culture

This study was approved by the Institutional Review Board of The First Hospital, Shanxi Medical University, Taiyuan, P.R., China. All participants provided written informed consent prior to their participation. For participants lacking mental or physical capacity to consent, a legal proxy provided written informed consent on behalf of the participant.

The human glioblastoma multiforme cell lines U87 and U251 were obtained from the American Type Culture Collection (ATCC; Manassas, VA). The human anaplastic astrocytoma cell line SHG44 was purchased from the Cell Bank of Type Culture Collection of the Chinese Academy of Sciences (Shanghai, China). The cells were cultured in Dulbecco’s modified Eagle’s medium (DMEM) supplemented with 10% fetal bovine serum (FBS) (Gibco/Invitrogen, NY, USA) at 37°C in a humidified incubator (CO_2_ water-jacketed incubator; Thermo Electron, Waltham, MA) under 5% CO_2_/95% air. Cells were fed every 3 days with complete medium and subcultured when 80% confluence was reached. Cultured primary astrocytes, used as a control, were obtained from a slightly impaired brain tissue fragment of a patient with intracerebral hemorrhage who consented to the procedure. The grey matter of the brain tissue was dissociated,washed in phosphate buffered sodium (PBS) and dispersed repeatedly. The resulting cell suspension was filtered and cultured in DMEM with 10% fetal bovine serum. After 2 weeks in culture, the remaining cells were mostly astrocytes [15].

### RNA Extraction and RT-PCR

According to manufacturers’ instructions, total RNA was extracted with TRIzol Reagent (Invitrogen, Carlsbad, CA, USA), and reverse transcriptase polymerase chain reaction (RT-PCR) was performed with a TaKaRa RNA PCR Kit (AMV) version 3.0 (TaKaRa, Dalian, China). The primers for human FRAT1 were: 5′-GCCCTGTCTAAAGTGTATTTTCAG-3′ and 5′-CGCTTGAGTAGGACTGCAGAG-3′; and the predicted PCR product was 325 bp. The primers for GAPDH, a housekeeping gene, which served as an internal control to normalize variances, were: 5′-GAAGTGAAGGTCGGAGTCA-3′ and 5′-TTCACACCCATGACGAACAT-3′; and the predicted product was 402 bp. All PCR reactions were performed using standard PCR conditions: 95°C for 5 min; 95°C for 1 min, annealing at 56°C for 1 min and extension at 72°C for 1 min for 30 cycles; and a final extension at 72°C for 10 min. The PCR products were electrophoresed in a 1.5% agarose gel containing 0.1 µg/µl ethidium bromide and visualized using a UV transilluminator (Alpha Innotech Corporation, San Leandro, CA, USA). Densitometric analysis was performed using Scion Image software (Scion Corporation, Frederick, MD, USA). A grayscale intensity value was determined for each target band.

### Protein Isolation from Cell Lines and Western Blot Analysis

Cells were harvested at the indicated time points, washed twice with ice-cold phosphate-buffered saline (PBS) (Gibco/BRL, Grand Island, USA) and lysed with cell lysis buffer on ice for 30 min. The supernatants were stored at 4°C after centrifuging at 12,000×g for 20 min, and total protein concentrations were determined using a BCA Protein Assay Kit (Pierce, Rockford, IL, USA). Equivalent amounts of total protein (40 µg/lane) were separated by 12% sodium dodecyl sulfate-polyacrylamide gel electrophoresis (SDS-PAGE), and then transferred to nitrocellulose membranes (Millipore, Bedford, MA, USA) in a transfer tank (Bio-Rad, California, USA) using the submerged method. After blocking for 2 h at room temperature in Tris-buffered saline (TBS; pH 7.4) with 0.1% Tween 20 (TBS-T) containing 5% nonfat dry milk, the membranes were incubated with goat anti-human IgG for FRAT1 (diluted 1∶200, Santa Cruz Biotechnology, CA, USA) as described previously [13]. Then, the membranes were washed extensively in PBS-T and incubated with horseradish peroxidase-conjugated secondary antibody (donkey anti-goat IgG, Santa Cruz Biotechnology, CA, USA) at room temperature for 1 h. The target bands were visualized using enhanced chemiluminescence (ECL) detection solution (Pierce, Rockford, IL, USA) and X-ray film (Eastman Kodak, Rochester, NY, USA). The membranes were reprobed with a β-actin mouse monoclonal antibody (1∶1000; Santa Cruz Biotechnology, CA, USA) to normalize for loading and/or quantification errors and to allow for comparisons of target protein expression. Protein expression was examined by densitometry using Scion Image software (Scion Corporation, Frederick, MD, USA).

### Plasmid Construction and Transfection

To study stable FRAT1 suppression, the pRNAT-U6.1/Neo plasmid (GenScript Corp., Piscataway, NJ, USA) which carries the green fluorescence protein gene, was used to express short hairpin RNAs (shRNAs) directed against FRAT1. Three FRAT1 target sequences (5′-GAGCTGGCAAGCAGGGCAT-3′, 5′-AGCTAGTGCTCTCTGGAAA-3′ and 5′-GCAGTTACGTGCAAAGCTT-3′) were selected for designing siRNA against FRAT1. A randomly scrambled sequence (5′-TTCTCCGAACGTGTCACGT-3′), which has limited homology to any known sequences in the human genome, was used as a negative control. To construct the shRNA plasmids, DNA oligonucleotides (Biomics Co., Ltd, Nantong, P.R., China.) containing the sense and the antisense siRNA sequences separated by a 9 bp spacer and having BamHI and HindIII compatible overhanging ends were annealed and ligated into linearized pRNAT-U6.1/Neo plasmid. All of the plasmids were confirmed by sequencing. The four resulting plasmids were designated as pRNAT-FRAT1-1, pRNAT-FRAT1-2, pRNAT-FRAT1-3 and pRNAT-NC, respectively. In initial experiments, pRNAT-FRAT1-3 plasmid was found to be the most potent for knockdown of FRAT1 (data not shown). Consequently, the pRNAT-FRAT1-3 plasmid was used to knock down FRAT1 expression in all subsequent experiments.

For gene transfection, 2×10^5^ U251 cells per well were plated onto 6-well plates and grown overnight to 60–70% confluence. Then, cells were transfected with pRNAT-FRAT1-3, pRNAT-NC and empty pRNAT-U6.1/Neo vector using Lipofectamine™ 2000 reagent (Invitrogen, Carlsbad, CA, USA) according to the manufacturer's instructions. Untransfected parental cells were used as a control for stable selection. Stable cell lines were selected with 800 µg/ml G418 (Sigma, St Louis, MO, USA), and resistant clones were pooled and cultured in medium containing G418. The stable clones were designated as U251-S (transfected with pRNAT-FRAT1-3), U251-neo (transfected with blank pRNAT-U6.1/Neo vector) and U251-NC (transfected with pRNAT-NC).

### MTT Assays

We performed MTT assays to determine the anti-proliferative effect of FRAT1 RNA interference (RNAi) on U251 cells. Both parental and transfected U251 cells in the log phase of growth were seeded in 96-well plates at a density of 1×10^4^ cells/well and cultured for 0, 24, 48, 72, 96, 120 and 144 h, respectively. Methyl thiazolyl tetrazolium (MTT; 10 µl of a 5 mg/ml solution; Sigma, St. Louis, MO, USA) was added, and cells were incubated at 37°Cfor an additional 4 h. The supernatant was aspirated gently, and the water-insoluble dark blue formazan crystals that formed during MTT cleavage in actively metabolizing cells were dissolved in 100 µl of dimethyl sulfoxide (DMSO; Sigma, St. Louis, MO, USA). The absorbance of each well was measured with a Bio-Rad 680 microplate reader (Bio-Rad, California, USA) at a wavelength of 490 nm. Cell growth curves were determined using the average absorbance at 490 nm from triplicate samples of three independent experiments.

### Cell Cycle Analysis by Flow Cytometry

Four cell lines were cultured in 25 ml flasks and incubated until they were 60–70% confluent in DMEM containing 10% FBS. The cells were collected and washed twice with ice-cold PBS, and then fixed overnight with 70% ethanol at 4°C. Following incubation with 50 µg/ml RNase A at room temperature for 30 min, the cells were stained with 20 µg/ml propidium iodide (PI; Sigma Co., St. Louis, MO, USA) for an additional 30 min. DNA content and cell cycle were analyzed by flow cytometry (FACSCalibur, Becton Dickinson, CA, USA), and the results were interpreted using Modifit and CellQuest software. All of the samples were assayed in triplicate.

### Plate Colony Formation Assay

Cells (1×10^3^) were plated in 10 ml of DMEM supplemented with 10% FBS and 800 µg/ml G418 in 60 mm dishes. After 2 weeks, colonies were rinsed with PBS, fixed with methanol for 5 min, and stained with Giemsa (Sigma Co., St. Louis, MO, USA) for 20 minutes. Clearly visible colonies (larger than 50 µm in diameter) were counted as positive for growth.

### Soft Agar Colony Formation Assay

Cells (1×10^4^) were added to 3 ml of fresh culture medium (DMEM supplemented with 10% FBS) with 0.3% agar and plated into 60 mm dishes that were previously covered with 6 ml of 0.5% agar medium. Cultures were maintained under routine conditions for 2 weeks, and colonies larger than 50 µm in diameter were counted as positive for growth under a microscope (Olympus, Tokyo, Japan). Assays were conducted in triplicate for three independent experiments.

### Monolayer Wound Healing Assay

Cells were cultured in 60 mm dishes containing DMEM with 10% FBS. The medium was replaced with FBS-free media and cells were cultured for 24 h to reach a final confluency of 90%. Monolayers of cells were scratched using a sterile 200 µl micropipette tip, rinsed several times with PBS to remove dislodged cells, and grown in FBS-free media for 24 h. The “wounds” were examined and photographed using a phase contrast microscope (Olympus, Tokyo, Japan) immediately after creating scratches and 24 h later. The migration distance in the wound was calculated by subtracting the distance between the lesions edges at 24 h from the distance measured at 0 h. Experiments were repeated three times in duplicate with comparable results.

### Transwell Matrigel Invasion Assay

Cell invasion in vitro was measured by using the Matrigel invasion assay as described previously [16]. Briefly, transwell insert chambers (Becton Dickinson, Franklin Lakes, NJ, USA) with 8 µm pore size filters were coated with a thin layer of Matrigel (Becton Dickinson, Bedford, MA, USA) on the upper surface of polycarbonate membrane, and cells were added at a density of 1×10^4^ cells/ml in DMEM supplemented with 200 µl serum-free DMEM. The chemoattractant of cell migration (500 µl DMEM with 10% FBS) was added into the bottom wells. Cells were allowed to invade Matrigel-coated inserts at 37°C for 24 h. After the medium was removed from upper chamber, the non-invaded cells on the upper surface of the filter were scraped off with a cotton swab. The cells that had migrated to the lower surface of the membrane were fixed with 4% formaldehyde for 15 min at room temperature. Then, the chambers were rinsed in PBS and stained with hematoxylin and eosin for 5 min. The membranes were excised from the insert and mounted onto glass slides for microscopic analysis. The numbers of migrated cells were counted at high-power magnification (×100) from six randomly selected visual fields of the filter. Experiments were carried out in triplicate on three separate occasions.

### In vivo Growth Assay

Twenty immune-deficient nude mice (BALB/c-nu; 4–6 weeks old; 16–18 g) provided by the animal center of the Fourth Military Medical University were randomly divided into 4 groups. The nude mice were maintained in pathogen-free environments. All animal experiments complied with the international guidelines for the care and treatment of laboratory animals. Equal numbers of U251, U251-neo, U251-NC and U251-S cells (1×10^7^) in logarithmic growth phase were harvested, washed in PBS, and resuspended in 200 µl of normal saline. Then the cell suspension was injected subcutaneously into the right flank tissue of nude mice to establish xenograft models. Over a 40-day observation period, nude mice were monitored daily, and the sizes of transplanted tumors were measured by slide caliper every 5 days. A growth curve for transplanted tumor was drawn after calculating the tumor volume by the following formula: volume = L×S^2^×1/2, where L stands for the longest tumor diameter and S stands for the shortest tumor diameter of the two dimensions. Animals were sacrificed at the end of observation, and tumor weights were measured. The transplanted tumor specimens were immediately excised, fixed with 10% formalin and embedded in paraffin.

### Histopathology and Immunohistochemistry

Sections of formalin-fixed and paraffin-embedded samples from nude mice (4 µm thickness) were stained with hematoxylin and eosin using standard histopathological technique. The expression levels of FRAT1 were determined by immunohistochemical staining as described previously [13]. In brief, sections were incubated with the rabbit anti-human FRAT1 polyclonal antibody (1∶50 dilution; Santa Cruz Biotechnology, CA, USA) overnight at 4°C in a humidified chamber. Then, the primary antibody was detected using the appropriate labeled streptavidin–biotin (LSAB) kit (Maixin Biotechnology, Fuzhou, China) according to the manufacturer’s instructions. Finally, the slides were stained with DAB (Sigma, St. Louis, MO, USA), counterstained in hematoxylin and examined under a light microscope (Olympus, Tokyo, Japan).

### Statistical Analysis

The experiments were performed in triplicate and repeated three times independently. SPSS 16.0 (SPSS Inc, Chicago, IL, USA) was used for all statistical analysis. Data were expressed as mean ± standard deviation (SD). Comparisons among all groups were performed using one-way analysis of variance (ANOVA). Then, the Student–Newman–Keuls test (SNK test/q test) was used for comparison of differences between the two groups by ANOVA. Values of P<0.05 were considered statistically significant in all cases.

## Results

### Elevated Levels of Expression of FRAT1 in Human Malignant Glioma Cell Lines

**Figure 1:**
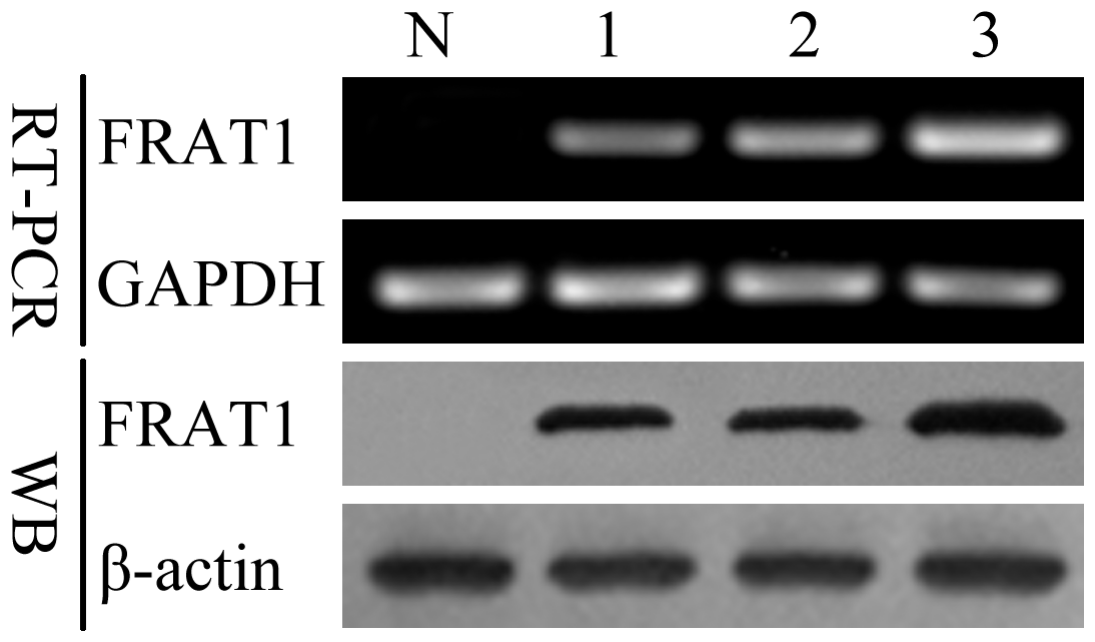
FRAT1 mRNA and protein levels in normal cultured primary astrocytes and SHG44, U87, U251 glioma cells as assessed by RT-PCR and Western blot analysis. For RT-PCR, specific FRAT1 primers yielded a 325-bp FRAT1 cDNA fragment, and internal control primers for GAPDH yielded a 402-bp GAPDH cDNA fragment. Western blot analysis (WB) of FRAT1 detected distinct bands with apparent molecular mass of 29 kDa. β-actin was assessed as a loading control. N: human normal astrocytes; 1: SHG44; 2: U87; 3: U251.

Expression levels of FRAT1 mRNA and protein in several high grade glioma-derived cell lines cultured in vitro, including SHG44, U87, and U251, were compared to expression levels in normal human cultured primary astrocytes (N) by RT-PCR and Western blot analysis (Fig. 1). The results showed that FRAT1 was highly expressed in the three glioma cell lines compared with the normal astrocytes, and that the expression level of FRAT1 was the highest in U251 cells. Based on the high levels of FRAT1, U251 cells were used as an appropriate in-vitro model for assessing FRAT1 function in subsequent experiments.

### Suppression of FRAT1 Expression in U251 Cells by RNAi

**Figure 2:**
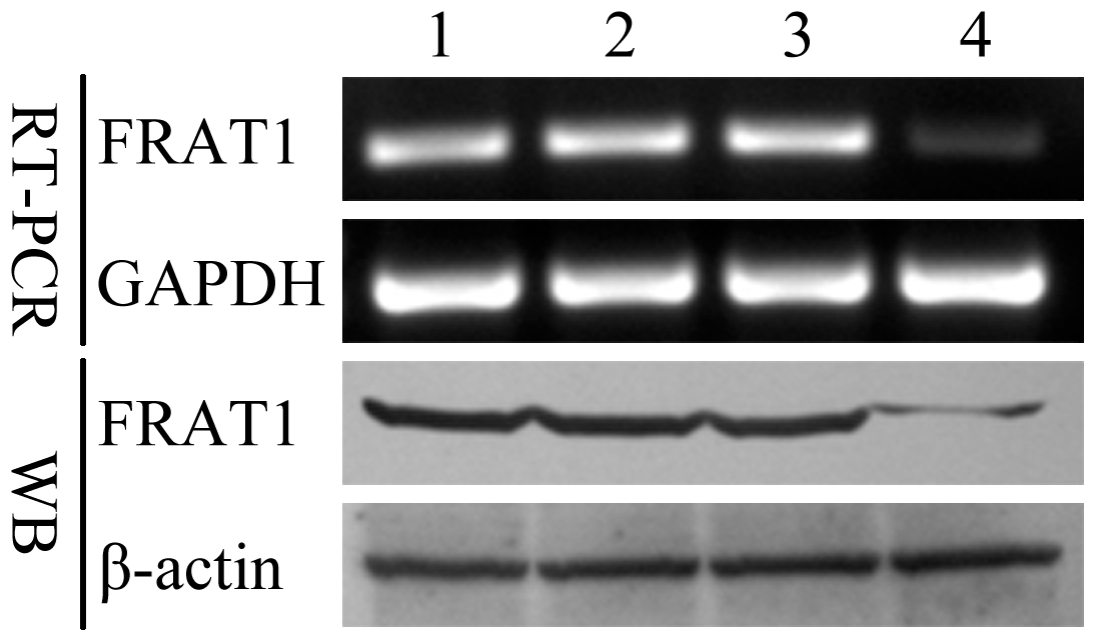
RNA interference reduced the expression of FRAT1 in U251 cells. Down-regulation of FRAT1 mRNA and protein expression in U251-S cells as compared to the parental U251, U251-NC, and U251-neo control cell lines was confirmed by RT-PCR and Western blot (WB). GAPDH was amplified as an internal control for the RT-PCR, and β-actin levels were examined as a loading control for the Western blot. 1: parental U251 cells; 2: U251-NC; 3: U251-neo; 4: U251-S.

To study the role of FRAT1 in the malignant progression of glioma, we established a stably transfected U251 cell line expressing shRNA against FRAT1. FRAT1 mRNA and protein expression was confirmed to be dramatically down-regulated in U251-S compared to parental U251, U251-NC, and U251-neo cells (P<0.05). In addition, there was no obvious difference of FRAT1 expression between the three control cell lines (P>0.05) (Fig. 2). These data indicate that the transfected FRAT1 shRNA significantly and specifically inhibits the endogenous FRAT1 expression in U251-S human glioma cells.

### Down-regulation of FRAT1 Inhibits U251 Cell Growth in vitro

**Figure 3:**
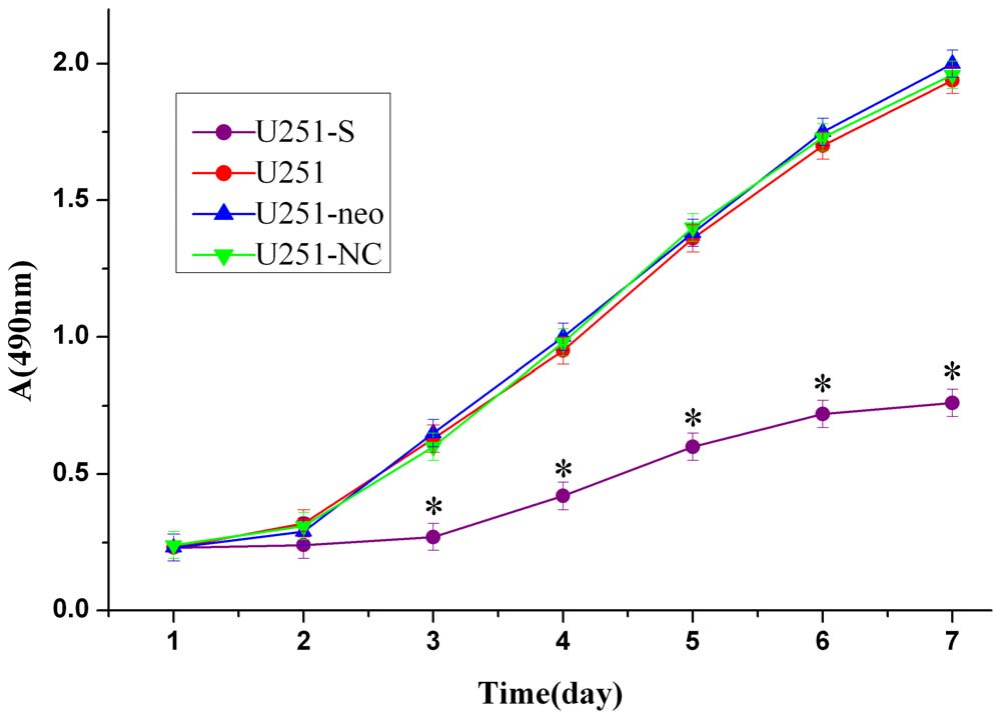
RNAi-mediated knockdown of FRAT1 inhibits growth of U251 cells in vitro. Cell viability was measured using a MTT assay. Cell growth curves were determined by absorbance at 490 nm. *, P<0.01 for U251-S relative to each of the three control lines.

To evaluate the effect of FRAT1 on the growth of U251 cells, viability curves for U251-S, U251, U251-neo, and U251-NC cells were determined by MTT assay. As shown in Fig. 3, the growth of U251-S cells was inhibited notably when compared with other groups, with this effect being most obvious from day 3 to day 7 (P<0.01). However, there were no significant differences in cell growth between the U251, U251-NC, or U251-neo cells (P>0.05). These results indicate that down-regulation of FRAT1 expression by RNAi markedly inhibits the growth of U251 cells.

### Knockdown of FRAT1 Inhibits Cell Cycle Progression of U251 Cells in vitro

**Figure 4:**
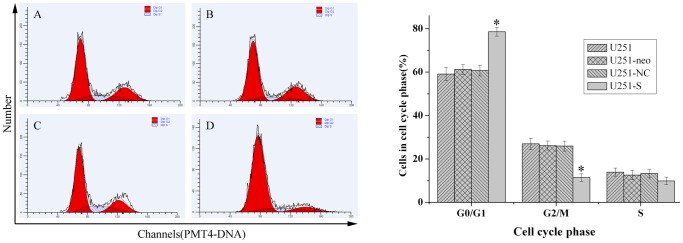
RNAi-mediated knockdown of FRAT1 affects the cell cycle distribution of U251 cells in vitro. *Left panel:* (A) parental U251, (B) U251-NC, (C) U251-neo and (D) U251-S were stained with propidium iodide. The DNA content and cell cycle were examined and analyzed by flow cytometry. *Right panel:* A histogram is provided showing the percentages of cells in each cell cycle phase as determined by gating of the flow cytometry.

To investigate the effect of FRAT1 knockdown on cell cycle progression, flow cytometry was performed to determine the cell cycle distribution. Compared with parental U251, U251-neo and U251-NC cells, U251-S cells accumulated in the G0/G1 phase (78.63±2.0%; P<0.01), whereas the percentage of cell numbers distributed in the G2/M phase were decreased sharply (11.45±1.8%; P<0.01 compared to any of the three control cells). There was no obvious difference in cell cycle distribution between parental U251, U251-neo or U251-NC cells (P>0.05; Fig. 4). These results suggest that reduction in FRAT1 expression in U251 cells by RNAi delays cell cycle progression and decreases cell proliferation.

### Knockdown of FRAT1 Inhibits Colony Formation in vitro

**Figure 5:**
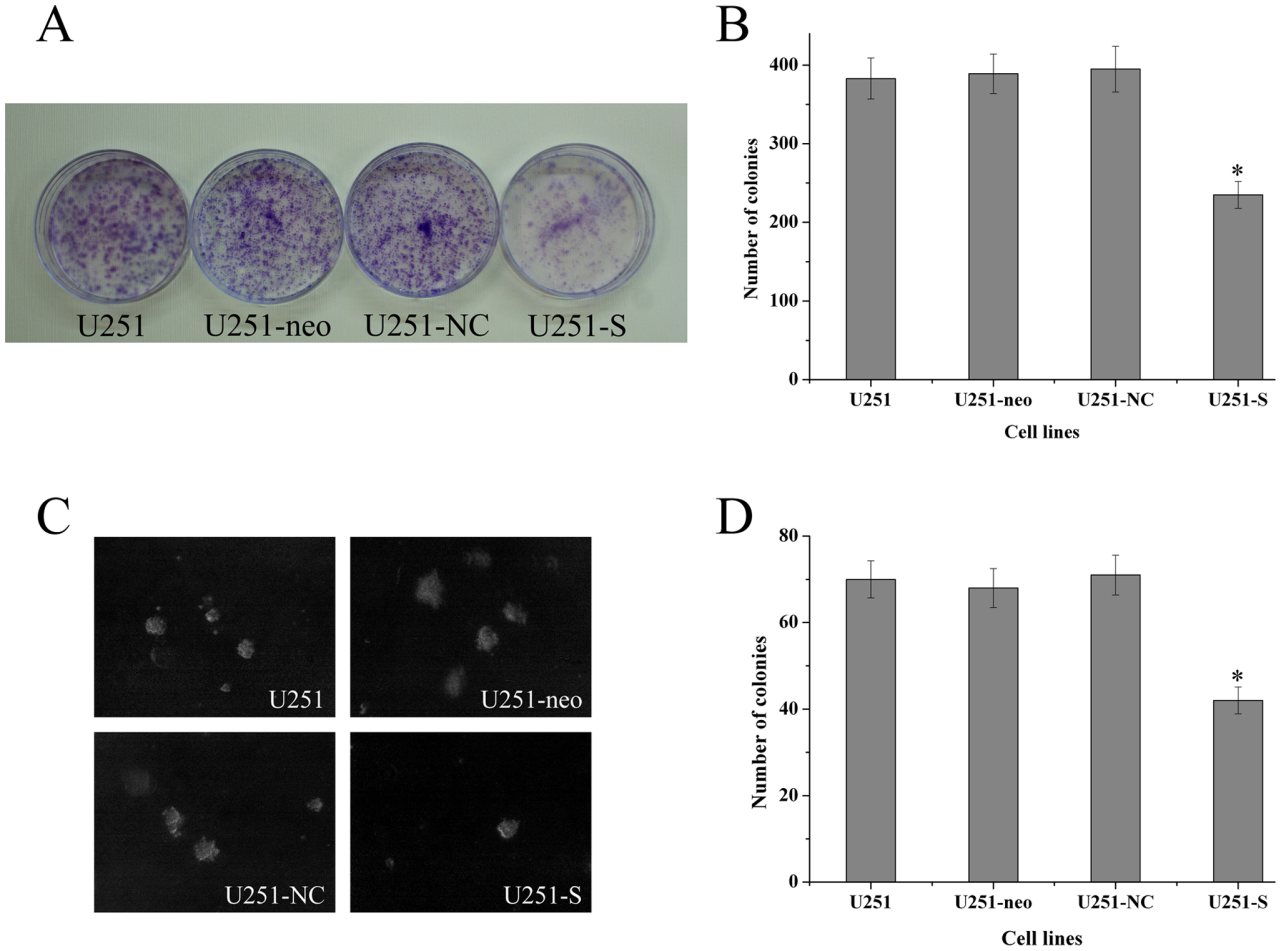
FRAT1 knockdown suppresses plate colony formation and soft agar colony formation. (A, B) Equal numbers of parental U251, U251-neo, U251-NC and U251-S cells were seeded onto 60 mm dishes. After 14 days, the cells were fixed and stained with Giemsa (A). The average number of colonies formed in three independent experiments was quantified (B). (C, D) Equal numbers of U251, U251-neo, U251-NC and U251-S cells were plated in 0.3% soft agar and cultured for 14 days. Colony formation was photographed under the microscope (C) and scored (D).

To analyze the effect of FRAT1 down-regulation on the anchorage-dependent growth potential of U251 cells, plate colony formation assays were performed for parental U251, U251-neo, U251-NC and U251-S cells. Compared to the control cells, the number and size of colonies for U251-S cells were significantly decreased (P<0.01). In contrast, There were no obvious differences in colony-forming ability among the three control lines (P>0.05; Fig. 5A and B). Furthermore, we evaluated the effect of FRAT1 knockdown on anchorage independent colony formation in soft agar. Similar findings were observed (Fig. 5 C and D). Taken together, these data indicate that reduction in FRAT1 expression decreases the colony-formation ability of U251 cells in vitro.

### Down-regulation of FRAT1 Inhibits the Migration of U251 Cells

**Figure 6:**
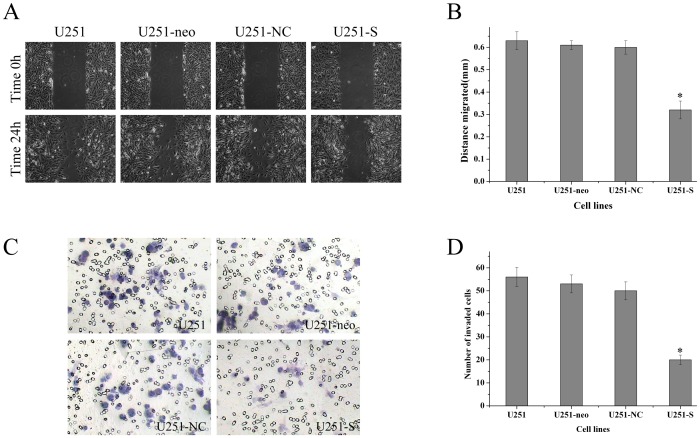
FRAT1 knockdown modulates migration and invasion of U251 cells. (A) FRAT1 knockdown inhibits cell migration. Monolayers of U251, U251-neo, U251-NC and U251-S cells were mechanically wounded with a pipette tip. Repair of the lesion by cell migration was photographed 24 h later. (B) The cell migration was quantified as shown. (C) FRAT1 RNAi diminished cell invasion of U251 cells. Each of the indicated U251 cell types was assayed for cell invasion using transwell tissue culture dishes. (D) The average cell counts of invading cells from 6 high power fields are shown.

To study whether FRAT1 suppression could influence migration of U251 cells, we used a wound healing assay to test parental U251, U251-neo, U251-NC and U251-S cells. As shown in Fig. 6 (A and B), the migration distances were 0.63±0.04 mm, 0.61±0.02 mm, 0.60±0.03 mm and 0.32±0.04 mm, respectively. These results demonstrate that the migration of U251-S cells is notably reduced compared with other groups (P<0.01). There were no obvious differences in migration among parental U251, U251-neo, and U251-NC cells (P>0.05). Thus, inhibiting FRAT1 expression blocks the migratory ability of U251 cells.

### FRAT1 Silencing Decreases Invasion of U251 Cells

Invasive growth is an important biological feature of malignant glioma cells. To evaluate the role of FRAT1 in glioma cell invasion activity, a transwell assay was performed. Representative micrographs of transwell filters are shown in Fig. 6C, and the number of invading cells is quantified in Fig. 6D. The U251-S group (20±2.1) had notably fewer invading cells than in the parental U251 group (56±4.2), the U251-neo group (53±3.9) or the U251-NC group (50±3.9) (P<0.01). No significant differences were observed in the invasiveness of the three control cell lines (P>0.05). These results suggest that down-regulation of FRAT1 expression significantly reduces the invasive potential of U251 cells in vitro.

### Inhibition of FRAT1 Expression Suppresses Growth of Transplanted Gliomas In Vivo

**Figure 7:**
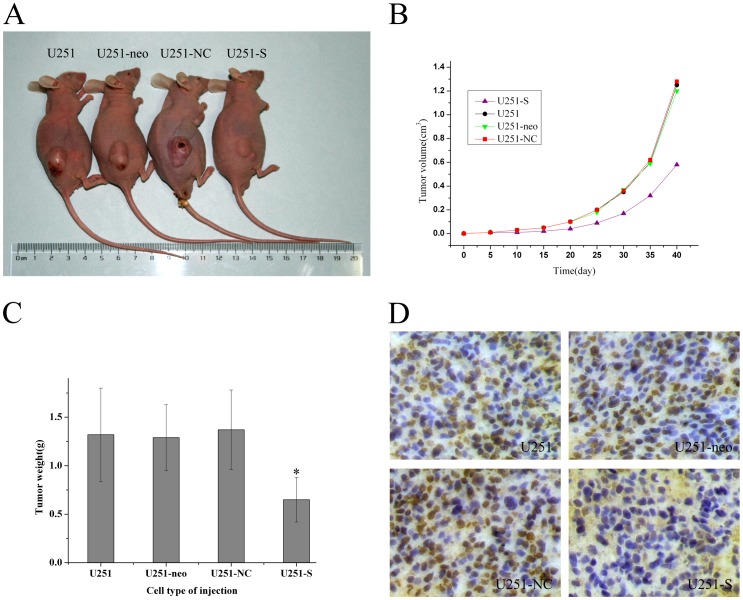
FRAT1 depletion decreases tumorigenicity in nude mice. (A) BALB/c-nu mice were injected subcutaneously with 1×10^7^ of U251, U251-neo, or U251-NC control cells; or U251-S FRAT1 knockdown cells. Representative tumor formation was photographed 40 days after injection. (B) Tumor sizes were determined by measuring the tumor volume every five days from 5 to 40 days after injection. (C) Average tumor weights of mice 40 days after injection are shown. Values represent means±SD obtained from three independent experiments. (D) Immunohistochemical analysis of FRAT1 expression in tumors in nude mice 40 days following injection.

We have demonstrated that FRAT1 depletion can efficiently inhibit cell proliferation, induce the G0/G1 cell cycle arrest and suppress the migration and invasion of U251 cells in vitro. To verify whether the effect of FRAT1 RNAi on growth of glioma cells is also observed in vivo, we injected parental U251, U251-neo, U251-NC or U251-S cells into nude mice to develop subcutaneous glioma xenografts. As shown in Fig. 7 (A, B), tumor growth was delayed for mice injected with U251-S cells, and the average tumor volume 40 days after transplantation (0.625±0.172 cm^3^) was significantly decreased compared with all other groups (P<0.01). There were no marked differences in tumor size among parental U251 (1.253±0.354 cm^3^), U251-neo (1.212±0.311 cm^3^), and U251-NC cells (1.301±0.326 cm^3^) (P>0.05). At 40 days, the mice were sacrificed and the weights of the tumors were recorded. Consistent with tumor volume outcome, the mean tumor weight of the U251-S group was prominently reduced compared to the control groups (Fig. 7C). The tumor weight showed no significant differences among parental U251, U251-neo, and U251-NC cells (P>0.05). In addition, staining results confirmed that FRAT1 expression was much weaker in transplanted tumor specimens derived from U251-S cells compared with the expression level of the other three groups, as assessed by immunohistochemical analysis (Fig. 7D). These data indicate that knockdown of FRAT1 expression markedly inhibits tumorgenesis in nude mice, and that FRAT1 RNAi can exert a strong antitumor effect on U251 cells in vivo.

## Discussion

Glioblastoma, which arises from neuroepithelial cells, is a challenging and highly invasive malignant neoplasm of the central nervous system. To date, the available treatments for glioblastoma offer only limited benefits. Despite recent advances in surgery and adjuvant therapy, patients with glioblastomas continue to have generally poor prognoses due to incomplete resection and resistance to radiotherapy and chemotherapy [17]. The underlying molecular mechanisms for the initiation and development of glioblastoma remain poorly understood [18]. Thus, the development of novel diagnostic and prognostic approaches to treat glioblastoma relies on a better understanding of the molecular pathogenesis of the glioblastoma. Identifying important molecular regulators of tumorigenesis, including those which regulate the invasive properties of glioblastoma, may provide potential targets for further therapeutic efforts.

The Wnt/β-catenin signaling cascade has been reported to be an evolutionarily conserved molecular pathway in metazoan animals. This pathway broadly modulates gene expression that governs embryogenesis and postnatal responses, such as cell proliferation, cell-fate determination, cell survival, cell behavior, and migration during morphogenesis [19]. Wnt/β-catenin signaling participates in the process of almost all aspects of neural development [20]. Consequently, upon dysregulated Wnt/β-catenin signaling, many human diseases ensue, including cancers (such as leukemia and colon cancer [21], [22]), osteoporosis, aging, and degenerative disorders [23], [24]. FRAT1 (also named GBP for GSK-3-binding protein), initially was identified in Xenopus as a protein that inhibits glycogen synthase kinase-3 (GSK-3) in vivo and appears to act as a positive regulator of the Wnt signaling pathway by stabilizing β-catenin [6], [25], [26]. Previous studies indicate that activation of the Wnt signaling cascade can cause recruitment of FRAT1 into the β-catenin degradation complex by Dishevelled (Dvl) family proteins, leading to the dissociation of GSK-3 from Axin and consequent inhibition of β-catenin phosphorylation [27], [28], [29], [30]. Cytoplasmic accumulation of unphosphorylated β-catenin may ensue. The increased concentration of β-catenin protein in the cytoplasm favors its translocation to the nucleus as a coactivator for the TCF/LEF (T-cell factor/lymphoid enhancer factor) family and activates the transcription of Wnt/β-catenin target genes, such as c-myc and cyclin D1, which function as oncogenes [31], [32], [33], [34]. The activation of oncogenes by the Wnt/β-catenin pathway is believed to contribute to tumor progression for a variety of cancer types. Thus it is not surprising that FRAT1 has been found to be strikingly overexpressed in several human cancers including esophageal cancer, cervical cancer, breast cancer, ovarian cancer, and non-small cell lung cancer [7], [10], [11], [12], [35]. We previously demonstrated that FRAT1 was overexpressed in all grades and most subtypes of resected glioma tested. Furthermore, in retrospective studies, the expression level of FRAT1 was positively correlated with increasing pathologic grade and glioma proliferation, and was negatively correlated with tumor apoptosis [13], [14]. In the current study, we confirmed that FRAT1 is overexpressed in three established glioma cell lines. Thus, the mechanistic function and expression pattern of FRAT1 suggests that it might be involved in tumorigenesis and malignant progression of glioma under certain pathological conditions. The apparent correlation of high levels of FRAT1 expression with the more advanced stages of glioma and other cancers is suggestive of its potential as a selective target for therapeutics; however, cell-specific targeting could also potentially be employed to provide additional selectivity.

RNAi, which was first discovered in *Caenorhabditis elegans* [36], is a gene silencing regulatory mechanism in most eukaryotic cells that serves to direct homology-dependent control of gene activity. RNAi technology has been proven to be a powerful experimental approach for selectively reducing target gene expression and has shown promising preclinical results in cell models and animal models to suppress neoplastic cell proliferation [37]. In this study, we used RNAi as a strategy to specifically knockdown FRAT1 expression in U251 glioblastoma cells to address whether RNAi-mediated inhibition of FRAT1 could regulate the biologic function of U251 cells. The expression of FRAT1 mRNA and protein was suppressed markedly by RNAi as assessed by RT-PCR and Western analysis. Moreover, FRAT1 knockdown caused a statistically significant reduction in cell viability and inhibited cell proliferation of U251 cells in vitro. Our results suggest that FRAT1 RNAi inhibits growth by arresting the cells in the G0/G1 phase of the cell cycle. These findings are consistent with our previous observation that enhanced levels of FRAT1 expression were positively correlated with higher proliferative activity in human astrocytoma [14]. In addition, the results of the monolayer wound healing assay and transwell invasion assay indicate that depletion of FRAT1 can significantly suppress migration and invasion of U251 cells in vitro. Our findings suggest that FRAT1 RNAi can also significantly suppress tumorigenesis and growth of transplanted U251-derived tumors in nude mice, and that this suppression may be associated with remarkable inhibition of proliferation and invasion of human glioma cells.

A previous study demonstrates that FRAT1 overexpression leads to aberrant activation of Wnt/β-catenin signaling in esophageal squamous cell carcinoma, and that FRAT1 can induce the expression of c-Myc which is a critical element in oncogenesis [35]. We also demonstrated that cytoplasm and/or nucleus accumulation of β-catenin is closely correlated with high FRAT1 expression in human gliomas [13]. However, little is known with regard to how FRAT1 regulates β-catenin in glioblastoma and whether c-Myc may be involved. FRAT1 could potentially regulate a number of other proteins and pathways in addition to the Wnt/β-catenin signaling pathway. Therefore, the exact underlying molecular mechanisms of this phenomenon in glioblastoma deserve further investigation.

Malignant tumors are known to be characterized by unlimited proliferation, migration and invasion. In the present study, we demonstrated that specific RNAi-mediated knockdown of FRAT1 effectively suppressed aspects of each of these tumorigenic properties of glioblastoma U251 cells, both in vitro and in vivo. Since key genetic, epigenetic and environmental factors associated with gliomagenesis remain incompletely defined, our findings not only provide more knowledge about the roles that Wnt/β-catenin pathway plays during the tumorigenesis of gliomas, but also contribute to a novel potential therapeutic strategy for treatment of patients with glioblastoma.

In summary, these observations, together with our previous study [13], [14], suggest that FRAT1 may play a pivotal role in the development and progression of gliomas. We propose that, based on both its expression pattern and its demonstrated functional role, FRAT1 may also be useful as a valuable biomarker for the molecular diagnosis of glioma and a promising candidate target for glioma therapy.
